# Single-tube, dual channel pentaplexing for the identification of *Candida* strains associated with human infection

**DOI:** 10.1038/s41598-019-51198-6

**Published:** 2019-10-11

**Authors:** Mohd Hanif Jainlabdin, Ambalika Batra, Edith Sánchez Paredes, Francisca Hernández Hernández, Guoliang Fu, Jorge Tovar-Torres

**Affiliations:** 1 Department of Biological Sciences, Royal Holloway University of London, Egham, Surrey United Kingdom; 2 Faculty of Nursing, International Islamic University Malaysia, Kuala Lumpur, Malaysia; 3 Faculty of Medicine, Universidad Nacional Autónoma de México, México, D.F., México; 4 Genefirst Ltd., Oxfordshire, United Kingdom

**Keywords:** Fungi, Molecular biology, Preclinical research

## Abstract

Invasive candidiasis is one of the most common nosocomial fungal infections worldwide. Delayed implementation of effective antifungal treatment caused by inefficient *Candida* diagnosis contributes to its notoriously high mortality rates. The availability of better *Candida* diagnostic tools would positively impact patient outcomes. Here, we report on the development of a single-tube, dual channel pentaplex molecular diagnostic assay based on Multiplex Probe Amplification (MPA) technology. It allows simultaneous identification of *C*. *auris*, *C*. *glabrata* and *C*. *krusei*, at species-level as well as of six additional *albicans* and non-*albicans* pathogenic *Candida* at genus level. The assay overcomes the one-channel one-biomarker limitation of qPCR-based assays. Assay specificities are conferred by unique biomarker probe pairs with characteristic melting temperatures; post-amplification melting curve analysis allows simple identification of the infectious agent. Alerting for the presence of *C*. *auris*, the well-characterised multi-drug resistant outbreak strain, will facilitate informed therapy decisions and aid antifungal stewardship. The MPA*-Candida* assay can also be coupled to a pan-Fungal assay when differentiation between fungal and bacterial infections might be desirable. Its multiplexing capacity, detection range, specificity and sensitivity suggest the potential use of this novel MPA-*Candida* assay in clinical diagnosis and in the control and management of hospital outbreaks.

## Introduction

Invasive fungal infections (IFIs) are a significant threat to immunocompromised patients^1^. The immune system of healthy individuals may also be compromised by severe health conditions such as burns, diabetes mellitus, organ transplantation, human immunodeficiency virus infections and disorders such as leukaemia and other malignancies. Invasive and aggressive medical practices and treatments such as surgery, the use of catheters, radiation and chemotherapy put patients at high risk from invasive fungal infections^2^. It is estimated that invasive fungal infections claim the lives of around 1.5 million people every year but epidemiological data are often negatively affected by challenges associated with laboratory diagnoses such as inefficient methodology and inadequate regional logistics^3^.

Among the prevalent fungal pathogens, species of the yeast *Candida* are a leading causes of invasive fungal disease and are commonly associated with nosocomial infections^4^. *Candida* species contribute 10–20% of hospital-associated infections^5^ and are responsible for 5–15% of bloodstream infections among immunocompromised and extreme age patients^6^. More than 90% of invasive candidiasis cases are caused by *C*. *albicans*, *C*. *glabrata*, *C*. *parapsilosis*, *C*. *tropicalis* and *C*. *krusei*^7,8^. In the United Kingdom, the most recent estimate of invasive candidiasis was 5,142 cases in 2013, with population incidence of 3.1 per 100,000 and 10.1 per 100,000 for candidemia and invasive candidiasis respectively^9^. Improving the diagnosis and management of candidiasis is essential for the timely prescription of correct therapeutic drugs to patients and the acquisition of reliable epidemiological data^3,10^. It is also important for the management and control of nosocomial outbreaks^11^.

Accurate identification of pathogenic *Candida* to species-level is key for effective treatment, as not all species are equally susceptible to individual antifungal drugs^12^. This is evidenced by intrinsic resistance of *C*. *krusei* to fluconazole and lower susceptibility of *C*. *glabrata* towards most antifungal drugs, including amphotericin B^13,14^. Along with species-level identification, the ability to detect other medically important species has become crucial as the emergence of non-albicans *Candida* species continues to rise. In recent years, multi-drug resistant *C*. *auris* outbreaks have been reported across major hospitals in the UK^15,16^. Public Health England Mycology Reference Laboratory has reported that all *C*. *auris* isolates in the UK are resistant to fluconazole, with variable resistance to polyenes (~20% for amphotericin B) and echinocandins (~10%)^17^. First reported in Japan as the cause of an ear infection^18^ *C*. *auris* has been shown to have a close genetic relationship with *C*. *haemulonii*. It is crucial therefore to differentiate between these two species for the efficient management and control of nosocomial *C*. *auris* outbreaks, as under such circumstances the isolation of carriers and the release of non-carriers is a clinical priority^16,19^.

Recent advances in the development of new and reliable methods of fungal nucleic acid detection hold promise for the early identification of fungal pathogens^20,21^. Conventional microbiological techniques such as blood cultures are positive in only 38–50% of proven invasive candidiasis^9,22^, while immunological detection of fungal cell wall markers suffer from low specificity and sensitivity^23^. Real-time PCR-based molecular methods such as Genesig kit (PrimerDesign), ViPrimePLUS *Candida albicans* qPCR kit (Vivantis) and Luminex xTAG Fungal Assay (Luminex Molecular Diagnostics) appear promising. However, these and similar products are often limited in the number of species that can be identified in a single assay and suffer from low multiplexing capacity.

Multiplex Probe Amplification (MPA) was developed as an alternative biomarker amplification method that overcomes such limitations^24^. It allows detection of multiple analytes in a single fluorescence detection channel; up to six probes with a unique melting temperature can be labelled with the same fluorophore and detected. Here we report on the development of MPA technology for identification of the main *Candida* strains that cause invasive candidiasis. As a pre-clinical, proof of concept study, an MPA*-Candida* assay was engineered to incorporate a pan-*Candida* biomarker probe able to identify at genus level the nine *Candida* species most frequently associated with candidiasis (*C*. *albicans*, *C*. *dubliniensis*, *C*. *tropicalis*, *C*. *parapsilosis*, *C*. *guilliermondii*, *C*. *haemulonii*, *C*. *krusei*, *C*. *glabrata* and *C*. *auris*). In addition, three species-specific biomarker probes were incorporated for the individual identification of multidrug-resistant *C*. *auris*, *C*. *glabrata* and *C*. *krusei*. The *in-vitro* characterisation of the MPA*-Candida* assay reported here highlights the potential of this novel molecular tool in clinical diagnosis of candidiasis, disease management, and in the control of nosocomial outbreaks. Informed prescription of the most suitable chemotherapy at the first time of asking will not only benefit patients but could also contribute to antifungal stewardship efforts worldwide.

## Results

### Identification and characterisation of fungal DNA biomarkers

**Figure 1:**
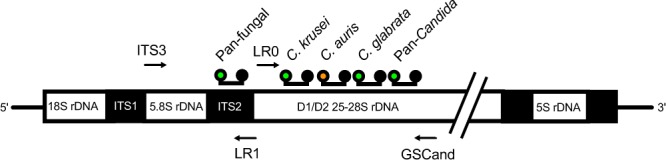
Relative position of amplification primers and probes along the fungal ribosomal genes. The relative location of two amplicons and five fungal biomarker target sequences are displayed. Pan-fungal primer set ITS3 and LR1 and pan*-Candida* primer set LR0 and GSCand are depicted as arrows. FAM-labelled pan-fungal, *C*. *krusei*-specific,* C. glabrata*-specific and pan*-Candida* probes are marked with a green circle; the HEX-labelled *C*. *auris*-specific probe is marked with an orange circle. Quencher molecules are shown as black circles. Not drawn to scale.

The basic principles of MPA technology are described and illustrated in the Methods section. Short biomarker nucleotide sequences that could reliably differentiate fungi from bacteria (pan-fungal), *Candida* from any other fungi (pan*-Candida*) and discern *C*. *auris*, *C*. *glabrata* and *C*. *krusei* from each other and from all other *Candida* species were identified within the rDNA locus^25–27^, as described in the Methods section. The pan-fungal probe lies within the internal transcribed spacer 2 (ITS2) region (Fig. 1). Reverse BLASTing of this sequence amongst the fungi demonstrated 100% sequence similarity with 100% query cover for all top 100 hits (Table S1). Optimised universal primers ITS3 and LR1 were used to generate an amplicon containing the pan-fungal biomarker. Amplicon sizes in the range of 279 bp (*Candida auris*) and 451 bp (*Lichtheimia corymbifera*, *formerly Absidia corymbifera*) were obtained.

Genus-specific (pan-*Candida*) and species-specific *Candida* biomarkers lie within the D1/D2 region of the LSU rDNA gene locus (Fig. 1). *C*. *krusei*, *C*. *glabrata* and *C*. *auris*-specific probes had 100% sequence similarity with 100% query cover to their respective *Candida* species (Table S1). The pan*-Candida* probe had 100% sequence similarity with all *Candida* species tested. Similarity to five other fungi rarely associated with human infection was also observed with this probe (Table S1). All selected biomarker sequences showed no intraspecies strain-to-strain variability in multiple alignments (Tables S2 and S3). A forward primer pair to amplify *Candida* species was optimised from universal primer LR0, while the reverse primer GSCand-R, was designed specifically for the assay (Table S4). The primer set generated a 381 bp biomarker-containing amplicon for most *Candida* species. *C*. *krusei*, *C*. *auris* and *C*. *guilliermondii* amplicons were 370 bp in size. Nucleotide sequences for all MPA-*Candida* probes and amplification primers are shown in Table S4.

### Design of *Candida*-specific MPA probes and melting curve analysis

**Figure 2:**
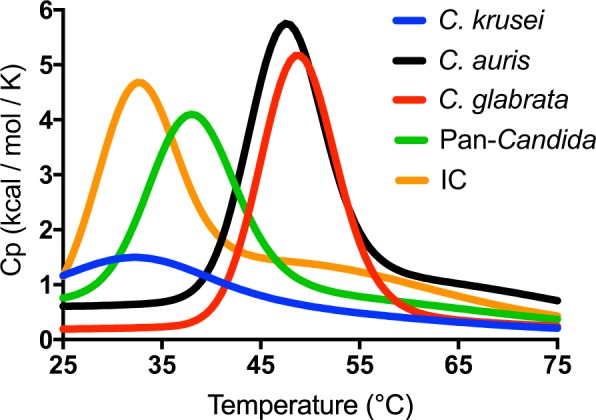
Theoretical T_m_ prediction for the MPA probes used in this study. Predicted melting curves for THO:PCO hybrids specific for *C*. *krusei*, *C*. *glabrata*, pan*-Candida*, *C*. *auris* and Internal Control (IC) probes using UNAFold software (http://unafold.rna.albany.edu/) are shown. Heat capacity (C_p_) is the maximum amount of heat needed to change the temperature of one mole of a hybrid by one degree at constant pressure, which defines the melting temperature (T_m_)_._

MPA probes consist of two partly complementary oligonucleotides, the dual-labelled target-hybridising oligonucleotide (THO) and a corresponding partially complementary oligonucleotide (PCO). Each THO is fully complementary to one of the *Candida*-specific biomarker target sequences identified above. The pan-*Candida*, and *C*. *krusei*, *C*. *glabrata* and *C*. *auris* species-specific probes hybridise with high affinity to their respective targets within the hypervariable D1/D2 LSU rDNA region. The PCO sequences were engineered to have several nucleotide mismatches against their corresponding THO sequence (Table S4). All *Candida* biomarker target sequences are located close to each other within the LSU region and are amplified by a single set of primers (Fig. 1). Initial characterisation of all five MPA probes (THOs plus their corresponding PCO sequences) using UNAFold software allowed confirmation of their theoretical melting profiles (Fig. 2). A melting curve overlap between the *C*. *krusei*-specific probe and the internal control probe as well as between the *C*. *auris* and the *C*. *glabrata*-specific probes is apparent. However, their independent analysis is not affected under experimental conditions as each overlapping probe is labelled with a different fluorophore. The pan-*Candida*, *C*. *krusei*-specific and *C*. *glabrata*-specific probes were labelled with 6-carboxyfluorescein (FAM) whereas the *C*. *auris*-specific and the internal control probes were labelled with Hexachloro-fluorescein (HEX).

**Figure 3:**
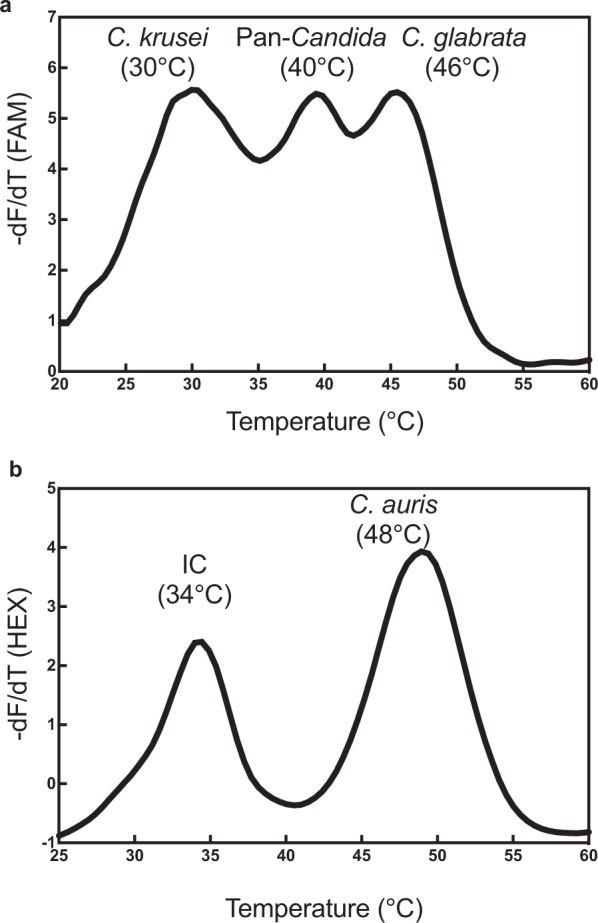
A two-channel MPA detection system with internal control and *Candida*-specific melt curve profiles. (**a**) The fluorescence negative derivative (−dF/dT) melt peaks obtained from dissociation between THO and PCO for the *C*. *krusei*-specific, pan*-Candida* and *C*. *glabrata*-specific probes in the FAM channel; (**b**) Melt peaks for the internal control (IC) and *C*. *auris*-specific MPA probes in the HEX channel. Their specific melting temperatures are indicated.

Experimental assembly of all MPA*-Candida* probes in a single reaction tube consistently generated five specific melt peaks collected in two fluorescence detection channels. *C*. *krusei*, *C*. *glabrata* and pan-*Candida* probes were detected in the FAM channel, whereas the *C*. *auris* and internal control probes were detected in the HEX channel (Fig. 3, panels a and b respectively). Each peak represents the maximum rate of dissociation between THO and PCO at a given temperature during melting curve analysis. These melt curves provide an important no-template control to compare against the melt curves obtained when target DNA is present and amplified in the reaction.

### Design of a complementary pan-Fungal qPCR assay

To complement the functionality of the MPA-*Candida* assay we designed a qPCR pan-Fungal assay that may be used alongside for enhanced fungal detection range. It operates under the same DNA amplification conditions and, when used, allows detection of a wider range of clinically relevant fungal pathogens. Fluorescence emission following qPCR biomarker amplification is monitored at 465 nm in the FAM channel. This reaction tube also contains a HEX-labelled internal control probe. Nucleotide sequences for the corresponding amplification primers and the pan-fungal probe are shown in Table S4.

### Analytical specificity of the combined MPA*-Candida* and pan-Fungal assays

**Figure 4:**
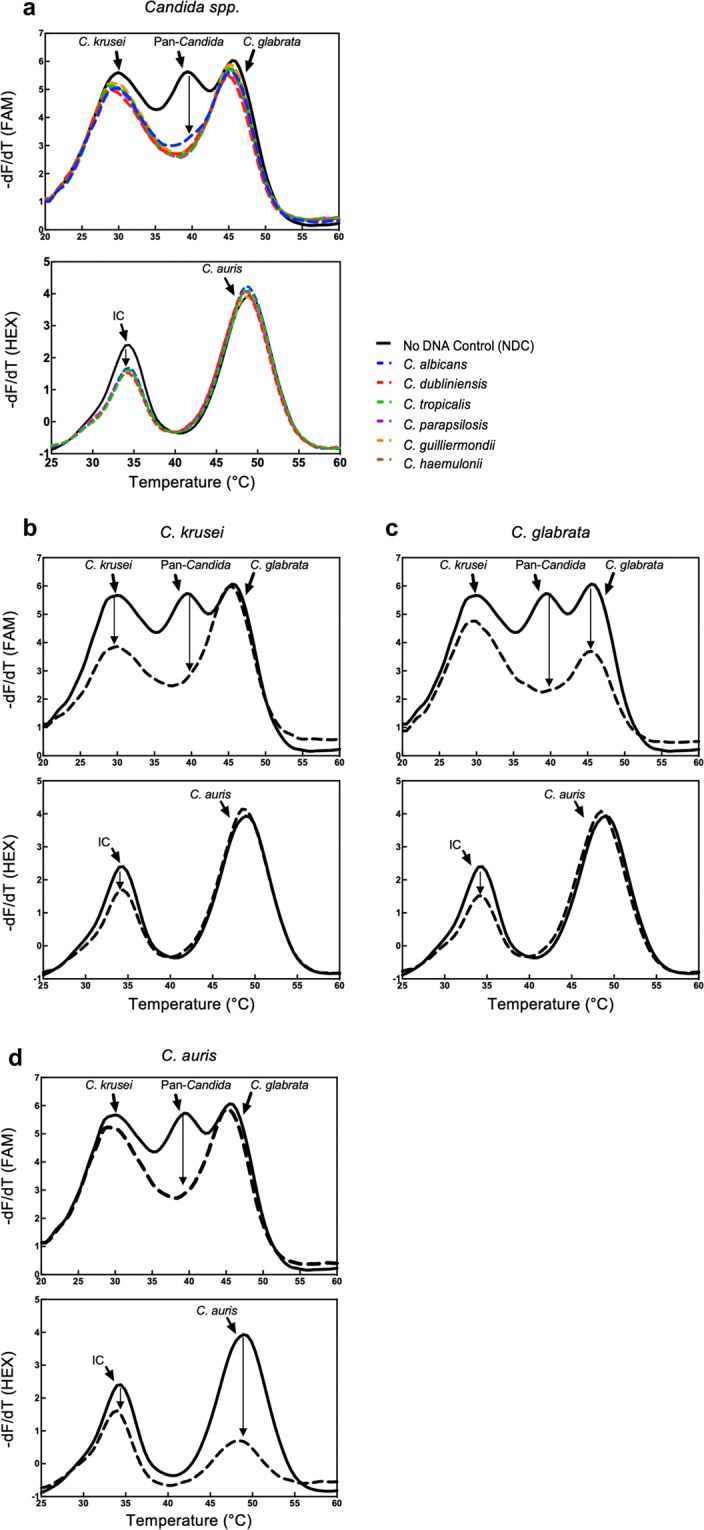
Melt curve analysis specificity of the MPA-*Candida* assay. FAM and HEX channel post-amplification melt curve profiles of nine different *Candida* species (dotted lines) compared to their corresponding no-template controls (solid lines). (**a**) Genus-level identification of the indicated *Candida* species is demonstrated by ablation of the pan*-Candida* peak in the FAM channel; decrease of the internal control (IC) peak in the HEX channel confirms assay functionality. (**b**,**c**) Species-specific identification of *C*. *krusei* and *C*. *glabrata* respectively, as demonstrated by a significant reduction in their corresponding melt peaks in the FAM channel; note that in both cases the pan-*Candida* peak is also ablated. (**d**) Species-specific identification of *C*. *auris*, as demonstrated by a significant reduction in its melt peak in the HEX channel and ablation of the pan-*Candida* peak in the FAM channel. In all cases diagnosis is supported by the corresponding DNA amplification curves and associated Ct values (Table S5).

The specificity of the MPA-*Candida* assay was tested independently using recombinant plasmids harbouring *C*. *albicans*, *C*. *dubliniensis*, *C*. *krusei*, *C*. *glabrata*, *C*. *auris*, *C*. *parapsilosis*, *C*. *tropicalis*, *C*. *haemulonii*, *C*. *guilliermondii* target biomarkers. Following DNA amplification their corresponding melt curves were analysed. All nine *Candida* species showed reduction/ablation of their melt peaks at 40 °C, corresponding to the pan-*Candida* probe, when compared to their corresponding no-template controls (Fig. 4). This demonstrates the assay’s capability to identify at genus level the nine *Candida* species most commonly associated with human infection. In addition, specific identification of three important drug-resistant *Candida* species, namely *C*. *krusei*, *C*. *glabrata* and *C*. *auris*, was demonstrated by additional melt peak reductions at 30 °C and 46 °C in the FAM channel and at 48 °C in the HEX channel respectively (Fig. 4, panels b–d). None of the other *Candida* species tested showed a melt curve decrease at those temperatures thus demonstrating the species-specificity of the assay for *C*. *auris*, *C*. *glabrata* and *C*. *krusei*.

Assay specificity was also tested using genomic DNA (Table 1). None of the heterologous DNAs tested were detected in the MPA*-Candida* assay; the internal control present in every sample demonstrated assay functionality and ruled out the possibility of false negative results. The pan-Fungal assay identified all nine *Candida* species tested as well as a range of pathogenic filamentous fungi, including *Aspergillus fumigatus*, *Fusarium solani*, *Penicillium rubens* and *Mucor circinelloides*. Genomic DNA from bacteria that form part of the normal human flora, including *Staphylococcus epidermidis*, *Staphylococcus aureus*, *Proteus mirabilis* and *Escherichia coli*, was not detected under the conditions used. All bacterial and fungal genomic DNAs were tested at 10^5^ genome equivalents (GEs) per reaction (Table 1; Fig. S1). DNA amplification of the internal control was consistently detected in the HEX detection channel. Early amplification (C = 16) of the same *E*. *coli* genomic DNA samples was observed using the universal bacterial 16S primer set.

**Table 1:** Specificity of the MPA*-Candida* and pan-Fungal assays.

Species	MPA-*Candida assay*							Pan-Fungal assay	
	Melting curve analysis					C_t_ value		C_t_ value	
	FAM			HEX		FAM	HEX	FAM	HEX
	CK	PC	CG	IC	CA			PF	IC
***Candida***									
*C*. *albicans*	−	+	−	+	−	23.40	26.00	22.00	21.00
*C*. *krusei*	+	+	−	+	−	22.20	26.20	22.90	25.60
*C*. *glabrata*	−	+	+	+	−	24.40	26.50	24.30	24.80
*C*. *auris*	−	+	−	+	+	23.60	29.00	23.00	26.20
*C*. *haemulonii*	−	+	−	+	−	24.60	27.80	21.00	26.00
*C*. *dubliniensis*	−	+	−	+	−	23.60	28.00	22.00	26.30
*C*. *parapsilosis*	−	+	−	+	−	23.90	26.90	21.80	25.40
*C*. *tropicalis*	−	+	−	+	−	23.50	26.00	21.00	26.00
*C*. *guilliermondii*	−	+	−	+	−	23.60	26.50	22.90	26.50
Moulds*									
*A*. *fumigatus*	−	−	−	+	−	−	28.10	22.90	23.40
*F*. *solani*	−	−	−	+	−	−	28.50	21.10	23.20
*P*. *rubens*	−	−	−	+	−	−	27.10	16.60	20.10
*M*. *circinelloides*	−	−	−	+	−	−	28.00	17.00	24.10
Bacteria*									
*S*. *aureus*	−	−	−	+	−	−	29.70	−	22.80
*S*. *epidermidis*	−	−	−	+	−	−	28.90	−	23.50
*P*. *mirabilis*	−	−	−	+	−	−	28.40	−	23.00
*E*. *coli*	−	−	−	+	−	−	28.00	−	23.20

*Fungal and bacterial genomic DNAs were tested at 10^5^ genome equivalent per reaction. C_t_: Threshold Cycle, the cycle intersection at the point where the curve first clearly rises off baseline value; FAM: Detection channel (465 nm/510 nm); HEX: Detection channel (533 nm/580 nm); CK: *C*. *krusei*-specific peak (30 °C); PC: Pan*-Candida* peak (40 °C); CG: *C*. *glabrata*-specific peak (46 °C); CA: *C*. *auris*-specific peak (48 °C); IC: Internal Control-specific peak (34 °C); PF: pan-fungal; +: Change in melt curve detected; −: No change detected.

### Assay sensitivity and limits of detection

**Figure 5:**
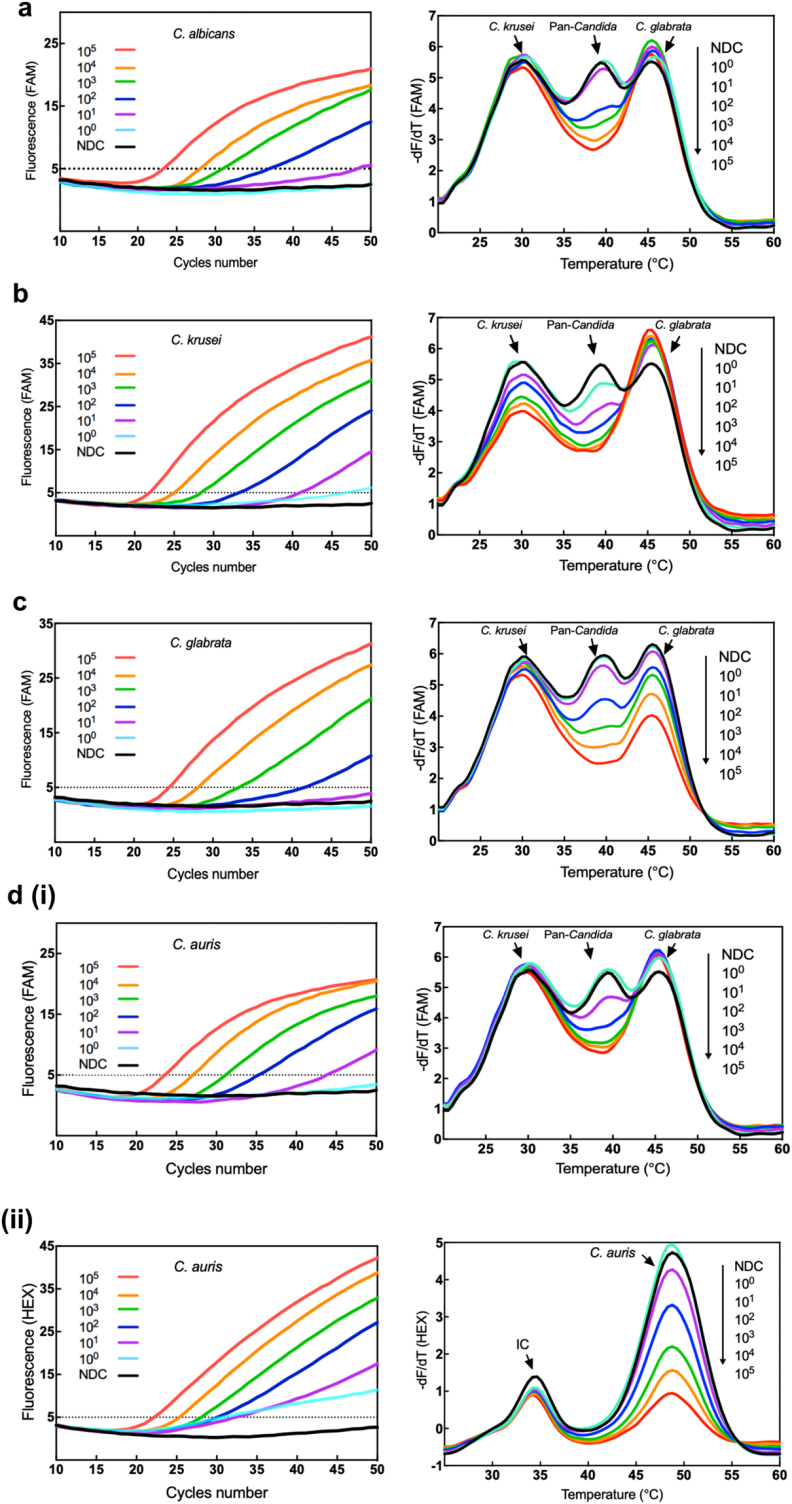
Amplification profiles and melt curve analyses of genomic DNA in MPA-*Candida* assays. Amplification reactions containing a range of 1 to 10^5^ GEs per reaction were set up. Post-amplification melt curve analysis was then carried out. Amplification plots of *C*. *albicans* **(a)**, *C*. *krusei* **(b)** and *C*. *glabrata* **(c)** and their corresponding melt curve profiles are shown demonstrating changes in the pan*-Candida* peaks proportional to the number of GEs present in the reaction. Similar results can be seen for *C*. *krusei* and *C*. *glabrata* at their corresponding species-specific peaks in the FAM channel. (**d**) Amplification plots of *C*. *auris* and their corresponding melt curve profiles showing proportional changes to their pan*-Candida* (**i**) and *C*. *auris*-specific (**ii**) peaks in the FAM and HEX channels respectively. The horizontal dotted line is fixed as the threshold value of fluorescence.

The limits of detection of the MPA*-Candida* assay were determined using a range of 10^0^–10^5^ GEs. DNA amplification profiles and the magnitude of melting curve reductions observed were evaluated against their corresponding no-DNA controls (NDC; Fig. 5). The amplification cycle threshold was set at 45 cycles to avoid inconclusive late amplification. Crucially, in this assay, interpretation of the specificity of amplification relies totally on the melt curve analysis derived from the data collected in just two detection channels, FAM and HEX.

Measurable reductions in the pan*-Candida* peaks compared to no DNA controls allowed genus-level identification of all samples, down to 10 GEs for *C*. *krusei* and *C*. *auris* and 100 GEs for *C*. *albicans*, *C*. *dubliniensis*, *C*. *glabrata*, *C*. *parapsilosis*, *C*. *tropicalis*, *C*. *haemulonii* and *C*. *guilliermondii* (Fig. 5 and Table 2). The *C*. *glabrata*-specific probe (46 °C peak in FAM channel) detected down to 100 *C*. *glabrata* GEs while the *C*. *krusei*- and *C*. *auris*-specific probes (30 °C peak in FAM channel and 48 °C peak in HEX channel) detected down to 10 GEs, a 10-fold higher sensitivity compared to the *C*. *glabrata* probe (Fig. 5, panels a to d).

**Table 2:** Limits of detection of the MPA*-Candida* assay.

Species	Detection channel(s)	Limit of detection (GEs)	C_t_ <45 cut-off value
*C*. *auris*	FAM, HEX	10	43.60, 32.40
*C*. *krusei*	FAM	10	41.00
*C*. *glabrata*	FAM	100	41.30
*C*. *albicans*	FAM	100	37.10
*C*. *dubliniensis*	FAM	100	35.60
*C*. *parapsilosis*	FAM	100	36.50
*C*. *tropicalis*	FAM	100	37.90
*C*. *guilliermondii*	FAM	100	36.50
*C*. *haemulonii*	FAM	100	37.00

The limits of detection of the pan-Fungal assay were determined from amplification curves monitored in the FAM channel. In general, *Candida* species can be routinely detected down to 10 GEs except for *C*. *glabrata*, *C*. *tropicalis* and *C*. *guilliermondii* which required a minimum of 100 GEs in the reaction. The limits of detection for both the MPA*-Candida* and pan-Fungal assays are summarised in Tables 2 and 3 respectively.

**Table 3:** Limits of detection of the pan-Fungal assay.

Species	Limit of detection (GEs)	C_t_ <45 cut-off value in FAM
*Aspergillus flavus*	10	40.00
*C*. *auris*	10	42.00
*C*. *krusei*	10	41.00
*C*. *albicans*	10	40.00
*C*. *dubliniensis*	10	41.30
*C*. *parapsilosis*	10	41.50
*C*. *haemulonii*	10	40.00
*C*. *glabrata*	100	43.10
*C*. *tropicalis*	100	35.00
*C*. *guilliermondii*	100	37.30

### Amplification efficiency and reproducibility of the pan-Fungal assay

**Figure 6:**
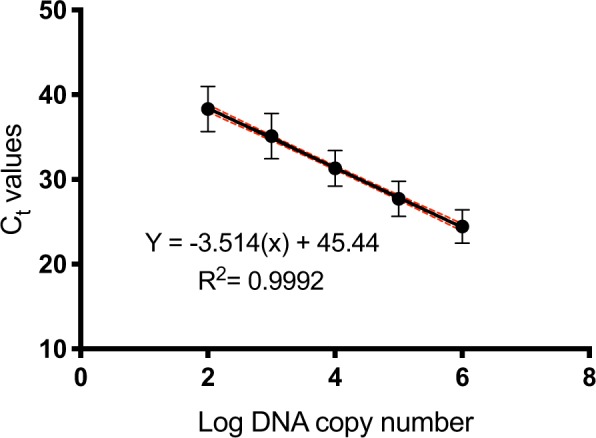
Pan-Fungal assay standard curve. Standard curve generated by plotting the mean C_t_ values as a function of target DNA concentration. Ten-fold serial dilutions (10^6^ to 10^2^ GEs) of *Candida* DNA were analysed in three independent experiments, each with three replicates per sample. Error bars represent standard deviations (SD) of C_t_ values. Confidence intervals (CI) of C_t_ mean values are indicated by the dashed lines.

Amplification efficiency of the pan-Fungal quantitative assay was calculated from the slopes generated in independent experiments using target biomarkers from eight *Candida* species. Standard curves were derived for each species in three independent experiments, each with triplicate samples incorporating a total of 360 data points. The efficiency range was 82.99 to 104.49% (Table S6), and the slopes between the standards were not significantly different (ANCOVA, p = 0.8561). A representative standard curve with an overall efficiency of 92.56% and correlation coefficient, R^2^ = 0.9992 is shown in Fig. 6.

Assay reproducibility was assessed using target biomarker dilutions spanning five orders of magnitude, from 10^6^ to 10^2^ plasmid DNA copies per reaction. The pan-Fungal assay reproducibly identified all *Candida* species over the range of dilutions tested, with intra- and inter-assay CV values lower than 5% (Tables S7 and S8 respectively).

### Comparison of assay performance

**Figure 7:**
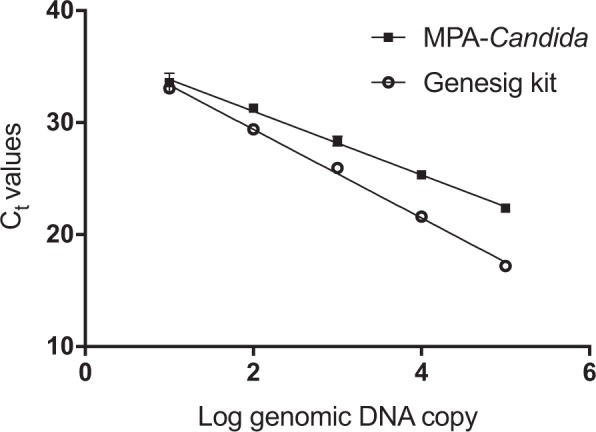
Detection of *C*. *auris* genomic DNA in MPA-*Candida* and simplex *C*. *auris* qPCR diagnostic kit (Genesig). *C*. *auris* genomic DNA was detected over five tenfold dilutions ranging from 10^5^–10^1^ GEs per reaction. Standard deviations are small and error bars can only be seen in the highest dilution C_t_ values.

Given the importance of multi-drug resistant *C*. *auris* for clinical and epidemiological studies, we compared the performance of the MPA-*Candida* assay against a commercially available single-purpose *C*. *auris* identification kit (Genesig, PrimerDesign). As can be seen in Fig. 7, comparable *C*. *auris* identification efficiency was observed under the same experimental conditions, with correlation coefficient R^2^ = 0.997, PCR efficiency (E) = 79.02% for the Genesig kit and R^2^ = 0.997, E = 125.09% for the MPA-*Candida* assay *C*. *auris* species-specific identification and R^2^ = 0.993, E = 73.57% for the *Candida* genus*-*specific identification respectively. In contrast to the Genesig kit, the MPA-*Candida* assay showed no cross-reactivity with genomic DNA from *C*. *haemulonii*, a close relative of *C*. *auris* (data not shown).

## Discussion

In this study, biomarkers for the detection of all fungal species (pan-fungal), all *Candida* species (pan-*Candida*) and the species-specific identification of *C*. *krusei*, *C*. *glabrata* and *C*. *auris* were incorporated into a novel molecular diagnostic assay based on MPA technology. *C*. *auris*, *C*. *glabrata* and *C*. *krusei* were selected for species-level identification due to their well-known antifungal drug resistant phenotypes. In this version of the assay, detection range was widen by incorporating genus-level detection of six additional *Candida* species capable of causing human invasive candidiasis, namely *C*. *albicans*, *C*. *dubliniensis*, *C*. *tropicalis*, *C*. *guilliermondii*, *C*. *parapsilosis* and *C*. *haemulonii*. The MPA*-Candida* assay contains probe pairs from the variable D1/D2 domains of the LSU rDNA locus. The initial 381-nucleotide sequence from the 5′ end of the LSU region has been shown to contain great intra-genus sequence diversity for *Candida* species and has been used previously to distinguish at species-level all clinically relevant *Candida* species^28,29^. BLAST analysis of the pan*-Candida* probe sequence found no identity to any prokaryotic or eukaryotic gene sequences, with the exception of five environmental yeasts, namely *Yamadazyma olivae*, *Kregervanrija pseudodelftensis*, *Zygosaccharomyces rouxii*, *Spathaspora* sp and *Saccharomyces cerevisiae*, the latter phylogenetically related to *Candida*^30,31^. Cross-detection of *S*. *cerevisiae* by the pan-*Candida* probe is not of medical concern as invasive infections caused by *S*. *cerevisiae* are rare compared to those caused by *Candida* and standard antifungal therapy with fluconazole is efficient in controlling such infections^32,33^.

The pan-fungal probe used in this study was selected from the ITS2 region, which was amplified using the universal primer set ITS3 and LR1^27^. Although there is no single locus for global fungal identification, the whole ITS region (ITS1-5.8-ITS2) is generally accepted as a universal fungal DNA barcode^26^. To ensure assay functionality and avoid false negative results, a unique internal control probe sequence was designed and incorporated into both MPA*-Candida* and pan-Fungal assays. Monitoring the presence of the internal control plasmid sequence was independent of the presence or absence of fungal target sequences in the assay.

*In vitro* testing of the MPA*-Candida* assay demonstrated its capacity for genus-level identification of nine medically important *Candida* species using a pan*-Candida* probe i.e., *C*. *albicans*, *C*. *dubliniensis*, *C*. *parapsilosis*, *C*. *tropicalis*, *C*. *guilliermondii*, *C*. *haemulonii*, *C*. *krusei*, *C*. *glabrata* and *C*. *auris*. Drug-resistant species *C*. *krusei*, *C*. *glabrata* and *C*. *auris* can also be simultaneously identified at species level by their corresponding species-specific probes. This should allow informed decisions to be made regarding chemotherapy. For example, an infection caused by *C*. *albicans* would give a positive diagnosis through reduction of the pan-*Candida* melting peak (Figs 4a and 5a). The concomitant absence of any significant change in the species-specific melting peaks for *C*. *auris*, *C*. *glabrata* or *C*. *krusei* would suggest the use of first-line antifungals such as fluconazol or itraconazole, instead of alternative and more costly second line antifungals recommended for *C*. *auris* or *C*. *glabrata* infections (e.g., micafungin, caspofungin, amphotericin B). In addition, kingdom-level identification by the pan-Fungal assay should strengthen clinical decisions where the prokaryotic or eukaryotic nature of the infectious agent is uncertain.

Intra-specific variation within the fungal rDNA locus has been shown to be minimal^34,35^. This includes the ITS region and the 26S D1/D2 region (target site for the pan-*Candida* and species-specific probes). Irinyi et al (2015) conducted extensive nucleotide diversity (π) and polymorphic site proportion analysis using 176 fungal species with a minimum of two clinical isolates per species. Kurtzman & Robnett (1997) reported intra-specific variation within the ~380 bp *Candida* D1/D2 region to be in the range of 0 to 2 nucleotides. Analysis of the intra-specific variability within the nucleotide sequences targeted by the MPA-*Candida* diagnostic probes using nucleotide sequences from reference and multiple clinical isolates/strains for each of the *Candida* species used in this study found no evidence of intra-species variability in their target regions (Tables S2 and S3). For *C*. *auris*, we included representative sequences from the most important geographical clades (East Asia, South Asia, Africa and South America). Taken together, these findings suggest that most if not all natural clinical isolates/strains of a particular *Candida* species should be readily identified by the MPA-*Candida* assay described in this study.

Inter-species variability within the D1/D2 region of the large ribosomal subunit is, on the other hand, exquisite. It has been extensively employed in fungal taxonomical classification and in the molecular identification of mould and yeast species, including *Candida*^28,35^. Table 1 shows that genomic DNA from a range of moulds and bacteria frequently associated with human colonisation/infection is not detected by the MPA-*Candida* assay; neither is genomic DNA of human origin. Extensive sequence homology searches using non-redundant nucleotide sequence databases that include bacterial, human, mould and yeast DNA sequences found no identical target sequences other than those reported on Table S1. Taken together, these data strongly suggest that the possibility of false positive diagnostic results due to inter-species cross-reactivity is very low. It is acknowledged however that, as for every other molecular diagnostic test, ruling out the possibility of unintended assay cross-reactivity would require more extensive sampling across the taxonomic range.

The fact that five different MPA probes are labelled with either of two different fluorophores and analysed using two fluorescence detection channels provides a crucial advantage over other real-time qPCR diagnostic tests which use standard hydrolysis probes and are limited to one detection channel per biomarker^24^. The pan-*Candida*, *C*. *krusei*-specific and *C*. *glabrata*-specific probes were labelled with FAM; the *C*. *auris*-specific and internal control probes were labelled with HEX. Although MPA allows labelling of up to six different probes with the same fluorophore we sought to avoid analytical ambiguity by using two rather than a single detection channel. The *C*. *auris*-specific probe has a melting temperature almost identical to that of the *C*. *glabrata*-specific probe and would overlap significantly in a single channel; the internal control probe has a melting temperature that is less than 5 ºC different to the *C*. *krusei*-specific probe. Ideally, the melting temperature between the probes labelled with the same fluorophore should differ by 5–7 °C. During assay optimisation assembly of the pan*-Candida* and *C*. *glabrata*-specific probes labelled with FAM at equivalent concentrations led to a masking of the *C*. *glabrata* signal; reducing the pan*-Candida* probe concentration to 0.2 µM resolved this issue. The reason for this observation is unclear but probe interference in MPA assays has been noted before^24^.

A separate pan-Fungal assay could assist clinicians in ruling out non-fungal infections before proceeding with the diagnosis for candidiasis. A pan-Fungal assay complementary to the MPA-*Candida* assay was engineered using a pan-fungal probe that targets the whole ITS2 region. The ITS2 region has been widely used in fungal phylogenetic studies and in detecting fungal pathogens in a range of PCR platforms^36,37^. Detection channels and the reaction protocol for the pan-Fungal assay are consistent with those used in the MPA*-Candida* assay, which enables simultaneous running of both reactions and result analysis in a single run. As the pan-Fungal assay is not based on MPA technology only an amplification curve is needed to monitor target amplification. The pan-Fungal assay is more straightforward to analyse and could save the precious DNA material and cut the cost for unnecessary diagnosis processes. However, combining the current MPA-*Candida* and pan-Fungal assays into a single-tube, single-detection channel reaction might be feasible.

The MPA*-Candida* assay offers post-amplification melt peak analysis for identification of drug sensitive and drug resistant *Candida* species. In the presence of each target, the corresponding melting peak is either reduced in magnitude or ablated when compared with the no-template control. All nine *Candida* species were detected by the pan*-Candida* probe and each drug resistant species (*C*. *auris*, *C*. *glabrata* and *C*. *krusei*) was simultaneously identified by their corresponding species-specific probe. A slight reduction of the *C*. *krusei* melting peak was noted in the presence of non-*krusei Candida* DNA throughout this study. This phenomenon has been described as “shrunk neighbouring peaks” by Fu *et al*.^24^ and does not affect the diagnostic specificity and accuracy of the assay. In the presence of *C*. *krusei* DNA its corresponding melting peak is clearly reduced or ablated compared to a small reduction as in the presence of non-*krusei Candida* DNA. Overall, assay design and melting peak distribution along two detection channels allows easy identification of one or more *Candida*-specific targets in a single-tube format.

Both the MPA-*Candida* and pan-Fungal assays were shown to detect down to 100 target GEs and could confidently detect 10 *C*. *krusei* and *C*. *auris* GEs. This range of detection is equivalent to the clinical range of candidemia of 5 to 100 CFU/ml^38^ and compares well with other commercially available kits. For example, SeptiFast (Roche) could detect fungal DNA in a range of 30 to 100 CFU/ml blood^39^, and MycoReal *Candida* kit (Ingenetix) could detect from 5 to 10 CFU/ml blood^40^. In the MPA*-Candida* assay, amplification of DNA from any of the *Candida* species is detected in the FAM channel whereas amplification of *C*. *auris* DNA or the internal control DNA is detected in the HEX channel. Post-amplification melt curve analysis is essential for the qualitative identification of drug-sensitive or drug-resistant species in the MPA*-Candida* assay. Interestingly, although DNA amplification from a single copy of *C*. *krusei* genomic DNA could be detected (C_t_ = 42.52 ± 2.07) its corresponding melting peak was not reduced significantly compared to the no-DNA control peak. Result interpretation therefore must always be guided by the melt curve analysis, especially at very low infection loads. Sensitivity testing using plasmid-cloned target DNAs demonstrated that the MPA-*Candida* assay can reliably detect down to 100 copies of target DNA per reaction (Tables S7 and S8). Given that each fungal cell contains around 50 rDNA copies, 100 copies of target DNA is equivalent to a limit of detection of 2 GEs/2 CFUs. The data suggests that the molecular environment within the reaction tube is important in determining assay sensitivity.

The MPA*-Candida* assay was evaluated for its capacity to identify the presence of the multi-drug resistant, outbreak strain *C*. *auris*. Assay sensitivity was found to be up to 10 GEs per reaction at both genus- and species-level. Its limit of detection is comparable to that of the Genesig kit (PrimerDesign), a simplex *C*. *auris* identification kit, when tested over a five order of magnitude *C*. *auris* DNA dilution range (Fig. 7). Importantly, no cross-reactivity was observed with *C*. *haemulonii*, a closely related species that is frequently misidentified as *C*. *auris* by commercial biochemical-based yeast identification systems such as Vitek 2 (BioMérieux) and BD Phoenix (BD Diagnostics)^41^.

In summary, the MPA-*Candida* assay allows detection of five different targets in just two detection channels. The multi-drug resistant *C*. *krusei*, *C*. *glabrata* and *C*. *auris* strains can be efficiently discriminated from other *Candida* species by a straightforward melt curve analysis. These features would allow clinical decision making to be expedited so that the most efficient chemotherapy can be prescribed in a timely fashion, thus improving patient outcomes. The MPA-*Candida* assay is suitable for adaptation to other qPCR platforms (Qiagen’s Rotor-Gene Q 2-Plex HRM, Applied Biosystems 7500), which could allow integration into laboratories that operate under conditions of limited resources.

## Methods

### Multiplex probe amplification (MPA) probes and melting curve analysis

**Figure 8:**
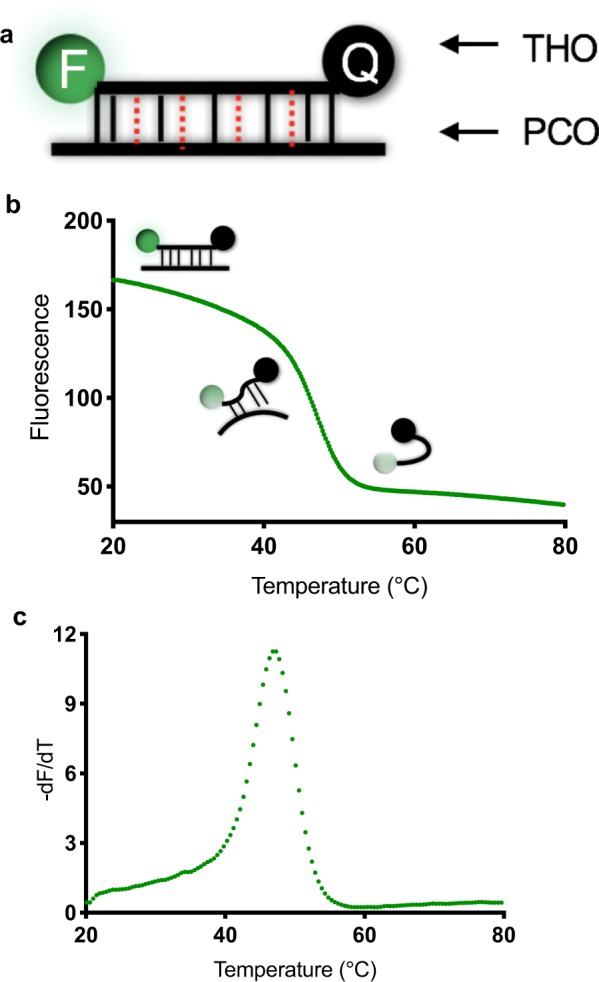
MPA technology principles: probes and melting curves. (**a**) Probes consist of a target-hybridising oligonucleotide (THO) labelled with a fluorophore (F) at the 5′end and quencher (Q) at the 3′end; the THO is shown hybridised to a partially complementary oligonucleotide (PCO). Mismatches are represented by red dotted lines. (**b**) In aqueous solution and low temperature the annealed THO: PCO pair is fluorescent due to the relatively high atomic distance between the fluorophore and the quencher. Exposure to increased temperature leads to thermal dissociation of the hybrid reaching a maximum rate at the corresponding dissociation temperature (T_m_). As the THO becomes detached, it assumes a randomly coiled conformation causing a drop in atomic distance between fluorophore and quencher and a corresponding decrease in fluorescence. Thus the melting curve shows decreased fluorescence emission with increasing temperature. (**c**) The negative derivative plot of the emission reading versus temperature reveals a positive value of the melting peak at the corresponding Tm.

Each probe for every target is composed of two oligonucleotides; a dual-labelled target-hybridising oligonucleotide (THO) and a partially-complementary oligonucleotide (PCO) (Fig. 8a). The THO binds to its intended biomarker target sequence with high affinity; it also binds to its corresponding PCO, albeit with lower affinity. The PCO incorporates mismatched nucleotides and has slightly lower annealing temperature compared to that of the THO and its fully complementary target sequence. In the absence of target DNA, the THO will bind to its corresponding PCO and will display a characteristic dissociation peak in the melt curve analysis. In the presence of DNA target sequence, the THO will preferentially anneal to its target and will be hydrolysed during the extension steps of PCR. Consequently, the post-amplification melting curve analysis will show a decreased melting peak compared to the no-target control as little or no THO:PCO hybrid can be formed. The THO:PCO hybrid probe shows maximum fluorescence at a low temperature which decreases as the probe dissociates with increasing temperatures (Fig. 8b). The highest rate of fluorescence change is observed in the melting curve analysis when 50% of the THO has dissociated from the PCO at the specific melting temperature (Fig. 8c)^24^.

### Fungal strains and bacterial isolates

Candida strains (*C. albicans, C. glabrata, C. tropicalis, C. dubliniensis, C. krusei, C. guilliermondii* and *C. parapsilosis*) were a kind gift of Professor Julian Naglik (King’s College London). *C. auris* (NCPF 8971) and *C. haemulonii* (NCPF 8402) strains were purchased from Public Health England, Bristol, UK. Mould and bacterial strains *A. fumigatus, F. solani, M. circinelloides, P. rubens, S. aureus, S. epidermidis, E. coli*, and *P. mirabilis* were obtained from the Molecular Microbiology laboratory, Centre for Biomedical Sciences, Royal Holloway University of London. Fungal isolates were sub-cultured onto Sabouraud-Dextrose Agar (SDA; Sigma-Aldrich) and incubated at 37° and 25 °C for yeast and mould respectively for 15–24 hours. For liquid cultures, single yeast colonies were inoculated in yeast-extract peptone dextrose (YPD) broth (Sigma-Aldrich), while bacterial isolates were cultured in Luria-Bertani (LB) broth (Merck) at 37 °C in a rotary shaker (SciQuip) for 24 hours. Following incubation, cultures were placed on ice and processed for DNA isolation immediately. The identity of all fungal isolates was confirmed by sequencing of their ribosomal DNA region and BLAST analysis (http://blast.ncbi.nlm.nih.gov).

### Nucleic acids extraction

One millilitre aliquots of yeast and bacterial cultures were transferred to 1.5 ml microcentrifuge tubes and centrifuged at 16,000 × g in a bench microcentrifuge for 2 minutes to pellet the cells. The supernatant was discarded and the bacterial and yeast cell pellets were processed as follows. The bacterial cell pellet was resuspended in 60 µl of 10 mg/ml lysozyme and incubated at 37 °C for 30 minutes to ensure efficient cells lysis. The yeast cell pellet was resuspended in 300 µl of lysis buffer from the Wizard Genomic DNA Purification kit (Promega). Following addition of 200 mg of 500-micron glass beads (BioSpec Products) yeast cells were homogenised with a FastPrep-24 instrument (MP Biomedicals) using five pulses with 60 seconds cooling intervals at 6.5 m/s speed setting. Cell debris was removed by centrifugation at 16,000 × g for 2 minutes, and the supernatant was transferred to fresh tubes for DNA isolation following the manufacturer instructions. Briefly, cellular proteins were removed by a salt precipitation step and the DNA was concentrated and desalted by isopropanol precipitation. The DNA was resuspended in 50 µl of DNA Rehydration Solution, a process optimised by incubating at 65 °C for 1 hour.

Cloned plasmid DNA was purified from bacterial cells using a QIAprep Miniprep Kit (Qiagen) according to the manufacturer instructions. Briefly, samples were subjected to alkaline lysis in buffer PB and neutralised. Cleared lysates were applied to QIAprep purification columns under high-salt concentration affinity binding conditions and eluted in 30 µl of elution buffer. In all cases, the concentration of purified DNA was measured using a NanoDrop 1000 spectrophotometer (Thermo Scientific) and stored at 4 °C until use.

### Primer and hydrolysis probe design

Fungal ribosomal DNA (rDNA) sequences were retrieved from GenBank and the International Society for Human and Animal Mycoses (ISHAM) ITS database and aligned using CLUSTALW2 or CLUSTAL Omega (EMBL-EBI; https://www.ebi.ac.uk/Tools/msa/clustalo). Sequences were manipulated and analysed using the BioEdit Sequence Alignment Editor Version 7.2.3. Primers and probes were designed within the rDNA region. Primers used in the MPA*-Candida* assay were the universal forward primer, LR0 and specifically designed reverse primer, GSCand. A universal pan-fungal primer set ITS3 and LR1 was selected to amplify the region containing the pan-fungal probe. The dual-labelled *Candida* probes were designed to specifically target the D1/D2 of the LSU rDNA region from at least 15 different *Candida* species. The pan-fungal probe was designed based on the internal transcribed spacer (ITS) sequences. MPA probes were designed using the nucleic acid folding and hybridisation prediction software UNAFold (http://unafold.rna.albany.edu). All primers and probes were subjected to reverse BLAST in GenBank (http://blast.ncbi.nlm.nih.gov) to test for cross-homology with other microorganisms. Universal bacterial 16S forward primer 516F: 5′-TGCCAGCAGCCGCGGTAA-3′ and reverse primer 806R: 5′-GGACTACCAGGGTATCTAAT-3′^42^ set was used for positive control in cross-reactivity experiments. MPA-*Candida* and pan-Fungal reaction sets are shown in Table 4.

**Table 4:** Description of the MPA*-Candida* and pan Fungal assays.

Reaction Type	Probe target	Detection channel	Primer set
MPA	*C*. *krusei*	FAM	Forward: LR0^43^ & Reverse: GSCand (this study)
	Pan-*Candida*		
	*C*. *glabrata*		
	*C*. *auris*	HEX	
	Internal Control		Forward: M13 (−20) & Reverse: M13 (−29)
qPCR	Pan-Fungal	FAM	Forwards: ITS3^44^ & Reverse: LR1^45^
	Internal Control	HEX	Forward: M13 (−20) & Reverse: M13 (−29)

FAM: 6-carboxy-fluorescein; 465 nm/510 nm; HEX: Hexachloro-fluorescein; 538 nm/580 nm. For simplicity and flexibility, the MPA*-Candida* assay was engineered as a two-tube system, run simultaneously under the same experimental conditions.

### MPA*-Candida* and pan-fungal real-time qPCR assay

The amplification assay was carried out in a LightCycler 480 Instrument II Real-time PCR unit (Roche Diagnostics) using a 384-well plate format. Amplification reactions were performed in 20 µl final volume reactions containing 2× LightCycler 480 Probes Master (Roche Diagnostics), 0.6 µM each primer, optimised concentrations of 0.2 µM pan*-Candida* THO, 0.4 µM *C*. *krusei*-specific THO, 0.4 µM *C*. *glabrata*-specific THO, 0.4 µM *C*. *auris*-specific THO and 0.4 µM internal control THO. Each THO was combined with the corresponding PCO at a ratio of 1:2. As for the pan-Fungal assay, both the pan-fungal and internal control probes were present at a concentration of 0.4 µM. Template DNA included 1 µl of internal control plasmid DNA (10^6^ copies) and 1 µl of either cloned (plasmid) or genomic fungal DNA (1 to 10^5^ copies/GEs) per reaction. Real-time PCR conditions were as follows: an initial step of 9 min 30 sec at 95 °C, followed by 50 cycles at 95 °C for 20 sec, 62 °C for 30 sec, and 72 °C for 1 min and temperature maintained at 95 °C for 10 sec before melting curve profiling increases from 20 °C to 80 °C. Two detection channels, FAM (465 nm/510 nm) and HEX (533 nm/580 nm) were activated for fluorescence measurements during the read steps at 72 °C for each experiment. The specificity of the pan-fungal probe was evaluated from the amplification curve whereas those of *Candida* MPA probes were evaluated from their melt curve profiles. The amplification curve threshold cycle (C_t_) of the pan-Fungal reaction was standardised by an automatic threshold setting whereas for MPA*-Candida* reaction the threshold line was set at 5 in Fit Points analysis method using the LightCycler 480 software version 1.5 (Roche Diagnostics). Both, the MPA and pan-Fungal reactions contain an internal control to ascertain assay functionality. The internal control probe consists of a unique nucleotide sequence cloned into the pCR 2.1-TOPO vector that has no identity to any entry in the non-redundant nucleotide databases at NCBI. To further demonstrate assay specificity, each probe was evaluated for detection of either genomic fungal DNA or plasmid DNA carrying the corresponding target sequence. In order to prepare cloned plasmid DNAs, first-round PCR amplicons of the *C*. *albicans*, *C*. *dubliniensis*, *C*. *parapsilosis*, *C*. *tropicalis*, *C*. *glabrata*, *C*. *guilliermondii*, *C*. *auris* and *C*. *krusei* were cloned into the pCR 2.1-TOPO vector using the TOPO TA Cloning system (Invitrogen). The resulting plasmid with insert was quantified and sequenced to confirm amplicon identity.

### MPA*-Candida* melting curve qualitative analysis

MPA*-Candida* assay relies on a novel melting curve analysis for multiple targets in one reaction. Dual-channel melting curves were obtained by measuring the fluorescence of the labelled THOs at different temperatures (from 20 to 80 °C). The fluorescence emission data was continuously collected at five acquisitions per degree Celsius and analysed by using the *Tm* Calling algorithm of the LightCycler 480 Software version 1.5 (Roche Diagnostics). Melting curves were displayed as fluorescence against temperature charts and plotted by representing the negative fluorescence derivative melting curve against temperature (−dF/dT). The point of inflection represents the melting point of the hybrid THO:PCO in the no DNA control reaction. In the presence of the target sequence, the corresponding probe will be hydrolysed during amplification, which will result in the appearance of a reduced peak in the post-amplification melt curve analysis. The no DNA control melt curve was used for comparison.

### Standard curve

Standard curve functions to assess unknown target copy number were developed using the pan-Fungal qPCR assay. Recombinant plasmid DNAs of *C*. *albicans*, *C*. *dubliniensis*, *C*. *krusei*, *C*. *glabrata*, *C*. *auris*, *C*. *parapsilosis*, *C*. *tropicalis*, *C*. *guilliermondii* were serially diluted for detection from 10^6^ to 10^2^ copies per reaction. Regression lines were obtained by plotting the logarithm of the initial plasmid copy number versus the corresponding C_t_ and used to determine the sensitivity and efficiency of the assay. Amplification efficiency (E) was calculated by E = 10^-1/b^− 1, where b is the slope of the linear regression equation.

### PCR assay reproducibility

Intra- and inter-assay reproducibility was assessed by two independent experiments of eight *Candida* species plasmid DNAs. The coefficient of variation (CV) calculated for C_t_ data was used as an indicator of relative precision and reproducibility. CV was determined by dividing the standard deviation (SD) by the arithmetic mean of the measured values: CV (%) = (SD/mean value) × 100.

## Supplementary information

Supplementary information

## Data Availability

All data generated or analysed during this study are included in this published article (and its Supplementary Information Files). Materials are available from the corresponding author on reasonable request.
