# Supplementary material for: Single-tube, dual channel pentaplexing for the identification of Candida strains associated with human infection
Source: Sci Rep. 2019 Oct 11;9:14692. doi: 10.1038/s41598-019-51198-6 (PMC6788996; doi:10.1038/s41598-019-51198-6)
Supplement: Supplementary file 1 — Supplementary information [file 41598_2019_51198_MOESM1_ESM.pdf]

SUPPLEMENTARY INFORMATION

Single-tube, dual channel pentaplexing for the identification of *Candida* strains associated with human infection.

Mohd Hanif Jainlabdin[1,2], Ambalika Batra[1], Edith Sánchez Paredes[3], Francisca Hernández Hernández[3], Guoliang Fu[4], Jorge Tovar-Torres[1]

[1]Department of Biological Sciences, Royal Holloway University of London, Surrey, United Kingdom, [2]Faculty of Nursing, International Islamic University Malaysia, Kuala Lumpur, Malaysia, [3]Faculty of Medicine, Universidad Nacional Autónoma de México, México D.F., México, [4]Genefirst Ltd., Oxfordshire, United Kingdom

SUPPLEMENTARY TABLES

Table S1. Homology- BLAST search for the pan-fungal and *Candida* probes.

Probe	Hits with 100 % sequence homology to probe / nature of organism	Other organisms with target sequences fully homologous to probe
Pan-fungal	100/100 fungi strains	None
Pan-*Candida*	38/38 *Candida* species strains	*Saccharomyces cerevisiae*<br>*Yamadazyma olivae,*<br>*Kregervanrija pseudodelftensis*<br>*Zygosaccharomyces rouxii*<br>*Spathaspora* sp.
C. krusei-specific	100/100 *C. krusei* strains	None
C. glabrata-specific	100/100 *C. glabrata* strains	None
C. auris-specific	90/90 *C. auris* strains	*Candida* sp. JHS-2008

Table S2. Intra-species conservation within the pan-*Candida* probe target region amongst clinical isolates and reference strains.

**Species**	**Number of strains**	**NCBI Accession number**	**Number of identical nucleotides in probe target region**
C. albicans	12	JX094791.1<br>KY106278.1<br>KC139704.1<br>KF418273.1<br>KJ624035.1<br>KJ624049.1<br>KY793751.1<br>KY793905.1<br>KT718116.1<br>KT718064.1<br>MH891781.1<br>KU987847.1	23/23
C. tropicalis	8	KY106850.1<br>MK394119.1<br>MH891783.1<br>AY951983.1<br>MK101221.1<br>U45749.1<br>MH748597.1<br>EF694617.1	23/23
C. parapsilosis	9	MH891788.1<br>EF694606.1<br>MK394125.1<br>KX792959.1<br>KU597281.1<br>U45754.1<br>AB199906.1<br>AB199909.1<br>MF767657.1	23/23
C. guilliermondii	5	MK101216.1<br>EU182216.1<br>U45709.1<br>KY108538.1<br>KT718072.1	23/23
C. dubliniensis	7	KJ451717.1<br>AB363780.1<br>KC146377.1<br>KF214388.1<br>KY106420.1<br>KJ624047.1<br>U57685.1	23/23
C. haemulonii	8	MK646002.1<br>KU883328.1	23/23

		KU896947.1<br>JX459762.1<br>KU883337.1<br>JN031572.1<br>JX459783.1<br>U44812.1	
Saccharomyces cerevisiae	4	ATCC204508<br>KY109314.1<br>MG641147.1<br>MH029910.1	23/23
Cryptococcus neoformans	4	KY107227.1<br>KY107211.1<br>L14068.1<br>AF356652.1	21/23
Cryptococcus gattii	3	FJ534907.1<br>KY107126.1<br>KY107041.1	21/23

Table S3. Intra-species conservation within the corresponding species-specific probe target regions amongst clinical isolates and reference strains.

**Species**	**Number of strains**	**NCBI Accession number**	**Number of identical nucleotides in probe target region**
C. krusei	9	KU904424.1<br>JN032661.1<br>KU729200.1<br>KP132510.1<br>KP132509.1<br>KF959838.1<br>KY108855.1<br>KY108853.1<br>KC601854.1	20/20
C. glabrata	11	JN032660.1<br>KY106475.1<br>KM103010.1<br>MH891784.1<br>MG859667.1<br>MH481612.1<br>KF214421.1<br>AB363784.1<br>KJ624034.1<br>KP780469.1<br>KP780472.1	23/23
C. auris	15	KC692056.1<br>KF689032.1<br>HE797774.1<br>JX459779.1<br>KJ126763.1<br>KF689036.1<br>KC692063.1<br>KF689037.1<br>KF689030.1<br>KF689031.1<br>KF689025.1<br>KF689035.1| KC692055.1<br>KJ126764.1<br>MK705920.1	27/27

Table S4. Nucleotide sequence and concentration of primers and probes used in the MPA-*Candida* assay.

Primer or probe	Sequence (5'- 3')	*Tm* (ºC)	µM[a]
MPA reaction probes			
C. krusei	THO:CAATCTACAACTCGTGCCGC	30 ±1	0.4
	PCO: GCGGCAtGAaTTaTAtATTG		0.8
Pan-*Candida*	THO:TACTTGTTCGCTATCGGTCTCTC	40 ±1	0.2
	PCO: GAGAtAtCGATAGCtAAtAAGTA		0.4
C. glabrata	THO:AACATAGGCAAAGTACAGTCCCA	46 ±1	0.4
	PCO: TGGGAaTGTAaTTTGaCTATaTT		0.8
C. auris	THO:TGGCCACCACGAGGTGTTCTAGCAGCA	48 ±1	0.4
	PCO: TGCTGCTAGActACtTCaTtGTGGCCA		0.8
Internal Control	THO:CTTATCGGAATAAGGGCGAATTCTGC	34 ±1	0.4
	PCO: GCAtAATTaGtCaTTATTaCtATAAG		0.8
MPA reaction primers			
Pan-*Candida*			
LR0-F	ACCCGCTGAACTTAAGCATA		0.6
GSCand-R	CTGTTTCACTCTCTTTTCAAAGT		0.6
Internal Control			
M13F	GTAAAACGACGGCCAG		0.6
M13R	GAAACAGCTATGACCATG		0.6
qPCR probes			

Pan-fungal	TATGCTTAAGTTCAGCGGGTA	0.4
Internal Control	CTTATCGGAATAAGGGCGAATTCTGC	0.4
qPCR primers		
Pan-fungal		
ITS3-F	GCATCGATGAAGAACGCA	0.6
LR1-R	GTTGGTTTCTTTTCCTCCG	0.6
Internal Control		
M13F & M13R	Same sequences and concentrations as in MPA reaction.	

[a] Final concentration used in each reaction

Lower case nucleotides in PCO sequence denote mismatch against THO; -F: forward; -R: reverse

Table S5. Specificity analysis of *Candida* DNA amplification threshold cycle (Ct values) in FAM and HEX channels.

***Candida* species**	**FAM**	**HEX**
	Mean Ct ± SD	Mean Ct ± SD
C. albicans	26.81± 0.16	31.85± 1.57
C. dubliniensis	22.60± 0.98	30.14± 0.60
C. tropicalis	25.75± 0.62	28.44± 1.44
C. parapsilosis	25.02± 0.10	28.48± 0.50
C. guilliermondii	26.67± 0.07	30.22± 0.57
C. haemulonii	25.60± 0.44	30.90± 0.04
C. krusei	25.08± 0.13	31.83± 0.39
C. glabrata	25.83± 0.48	29.08± 2.67
C. auris	24.23± 0.65	25.97± 0.04

Table S6. Standard curves for the pan-Fungal assay generated from three independent experiments for *Candida* DNAs within a range of 10^6 to 10^2 copies.

***Candida* species**	**Equation**	**R^2**
C. albicans	Y = -3.365*X + 46.37	0.9994
C. dubliniensis	Y = -3.393*X + 44.42	1
C. krusei	Y = -3.227*X + 45.11	0.9772
C. glabrata	Y = -3.811*X + 48.53	0.9891
C. auris	Y = -3.348*X + 41.79	0.9996
C. parapsilosis	Y = -3.633*X + 43.99	0.9957
C. guilliermondii	Y = -3.726*X + 47.61	0.9938
C. tropicalis	Y = -3.644*X + 45.89	0.9962

Table S7. Intra-assay reproducibility of the pan-Fungal assay for detection of *Candida* DNA

C. krusei

Log target copies/ PCR reaction	Ct 1	Ct 2	Ct 3	Mean Ct ± SD	% CV
10^2	35.58	34.07	35.99	35.21 ± 1.01	2.87
10^3	34.55	34.4	34.57	34.51 ± 0.09	0.27
10^4	31.84	31.79	31.56	31.73 ± 0.15	0.47
10^5	28.31	28.92	28.77	28.67 ± 0.32	1.11
10^6	24.86	23.66	24.81	24.44 ± 0.68	2.78

C. albicans

Log target copies/ PCR reaction	Ct 1	Ct 2	Ct 3	Mean Ct ± SD	% CV
10^2	37.76	36.98	37.9	37.55± 0.50	1.32
10^3	34.94	35.41	34.89	35.08± 0.29	0.82
10^4	32.73	32.78	32.76	32.76± 0.03	0.08
10^5	29.6	29.14	29.08	28.27± 0.28	0.97
10^6	25.85	25.17	25.73	25.58 ± 0.36	1.42

C. parapsilosis

Log target copies/ PCR reaction	Ct 1	Ct 2	Ct 3	Mean Ct ± SD	% CV
10^2	35.19	34.48	35.72	35.13 ± 0.62	1.77
10^3	32.86	31.77	32.24	32.29± 0.55	1.69
10^4	28.81	29	28.95	28.92± 0.10	0.34
10^5	24.88	24.82	24.87	24.86± 0.03	0.13
10^6	22.41	22.62	22.33	22.45 ± 0.15	0.67

C. guilliermondii

Log target copies/ PCR reaction	Ct 1	Ct 2	Ct 3	Mean Ct ± SD	% CV
10^2	37.51	38.21	37.63	37.78± 0.37	0.99
10^3	33.4	33.76	33.93	33.70± 0.27	0.80
10^4	30.92	30.74	30.84	30.83 ±0.09	0.29
10^5	27.97	27.94	27.39	27.77±0.33	1.18
10^6	25.5	25.5	25.82	25.61±0.18	0.72

C. auris

**Log target copies/ PCR reaction**	**Ct 1**	**Ct 2**	**Ct 3**	**Mean Ct ± SD**	**% CV**
10^2	35.36	34.58	35.01	34.98± 0.39	1.12
10^3	32.06	32.07	31.98	32.04± 0.05	0.15
10^4	28.57	28.51	28.54	28.54± 0.03	0.11
10^5	24.88	24.97	24.66	24.84± 0.16	0.64
10^6	20.73	21.21	21.01	20.98± 0.24	1.15

C. glabrata

**Log target copies/ PCR reaction**	**Ct 1**	**Ct 2**	**Ct 3**	**Mean Ct ± SD**	**% CV**
10^2	40.77	40.49	39.39	40.22± 0.73	1.81
10^3	37.04	36.74	37.6	37.13± 0.44	1.18
10^4	32.26	32.77	32.31	32.45± 0.28	0.87
10^5	29.44	29.11	29.17	29.24± 0.18	0.67
10^6	26.03	25.96	25.64	25.88± 0.21	0.80

C. tropicalis

**Log target copies/ PCR reaction**	**Ct 1**	**Ct 2**	**Ct 3**	**Mean Ct ± SD**	**% CV**
10^2	41.19	39.79	38.16	39.71± 1.52	3.82
10^3	35.33	35.45	35.7	35.49± 0.19	0.53
10^4	30.83	30.85	30.68	30.79± 0.09	0.30
10^5	26.96	27.16	27.07	27.06± 0.10	0.37
10^6	23.94	24.08	24	24.01± 0.07	0.29

C. dubliniensis

**Log target copies/ PCR reaction**	**Ct 1**	**Ct 2**	**Ct 3**	**Mean Ct ± SD**	**% CV**
10^2	38.1	38.26	37.41	37.92± 0.45	1.19
10^3	34.13	34.04	34.13	34.10± 0.05	0.15
10^4	30.97	31.06	31.09	31.04± 0.06	0.20
10^5	27.45	27.54	27.72	27.57± 0.14	0.50
10^6	23.58	23.46	23.56	23.53± 0.06	0.27

Table S8. Inter-assay reproducibility of the pan-Fungal assay for the detection of *Candida* DNA

C. krusei

Log target copies/ PCR reaction	Day 1			Day2			Mean Ct ± SD	% CV
	Ct 1	Ct 2	Ct 3	Ct 1	Ct 2	Ct 3		
10^2	35.58	34.07	35.99	37.47	36.65	38.74	36.42 ± 1.61	4.42
10^3	34.55	34.4	34.57	36.51	34.7	34.51	34.87 ± 0.81	2.32
10^4	31.84	31.79	31.56	30.9	31.11	30.33	31.26 ± 0.59	1.88
10^5	28.31	28.92	28.77	27.62	27.8	28.01	28.24 ± 0.53	1.86
10^6	24.86	23.66	24.81	23.01	23.42	23.92	23.95 ± 0.75	3.13

C. albicans

Log target copies/ PCR reaction	Day 1			Day2			Mean Ct ± SD	% CV
	Ct 1	Ct 2	Ct 3	Ct 1	Ct 2	Ct 3		
10^2	37.76	36.98	37.9	38.54	37.96	37.58	37.79± 0.51	1.35
10^3	34.94	35.41	34.89	34.63	32.68	35.16	34.62± 0.99	2.85
10^4	32.73	32.78	32.76	30.82	30.77	30.77	31.77± 1.08	3.40
10^5	29.6	29.14	29.08	27.53	27.71	26.19	28.21± 1.29	4.58
10^6	25.85	25.17	25.73	24.67	24.52	24.5	25.07 ± 0.61	2.42

C. parapsilosis

Log target copies/ PCR reaction	Day 1			Day2			Mean Ct ± SD	% CV
	Ct 1	Ct 2	Ct 3	Ct 1	Ct 2	Ct 3		
10^2	35.19	34.48	35.72	34.9	34.33	35.29	34.99 ± 0.52	1.49
10^3	32.86	31.77	32.24	31.31	31.37	31.28	31.81± 0.63	2.00
10^4	28.81	29	28.95	27.83	27.79	27.77	28.36± 0.62	2.18
10^5	24.88	24.82	24.87	23.87	23.8	23.61	24.31± 0.61	2.50
10^6	22.41	22.62	22.33	21.08	20.89	20.73	21.68 ± 0.86	3.98

C. guilliermondii

Log target copies/ PCR reaction	Day 1			Day2			Mean Ct ± SD	% CV
	Ct 1	Ct 2	Ct 3	Ct 1	Ct 2	Ct 3		
10^2	37.51	38.21	37.63	39.51	38.71	41.43	38.82± 1.47	3.79
10^3	33.4	33.76	33.93	34.29	34.79	34.74	34.15± 0.56	1.63
10^4	30.92	30.74	30.84	31.31	31.25	31.31	31.06 ±0.26	0.83
10^5	27.97	27.94	27.39	26.61	26.89	26.79	27.27±0.59	2.18
10^6	25.5	25.5	25.82	23.86	24.06	23.91	24.78±0.92	3.72

C. auris

Log target copies/ PCR reaction	Day 1			Day2			Mean Ct ± SD	% CV
	Ct 1	Ct 2	Ct 3	Ct 1	Ct 2	Ct 3		
10^2	35.36	34.58	35.01	34.85	34.32	35.23	34.89± 0.39	1.13
10^3	32.06	32.07	31.98	31.17	31.67	30.93	31.65± 0.49	1.55
10^4	28.57	28.51	28.54	28	27.99	27.85	28.24± 0.33	1.17
10^5	24.88	24.97	24.66	24.51	23.93	24.44	24.57± 0.37	1.52
10^6	20.73	21.21	21.01	20.78	21.19	20.95	20.98± 0.20	0.96

C. glabrata

Log target copies/ PCR reaction	Day 1			Day2			Mean Ct ± SD	% CV
	Ct 1	Ct 2	Ct 3	Ct 1	Ct 2	Ct 3		
10^2	40.77	40.49	39.39	38.49	39.87	43.51	40.42± 1.72	4.25
10^3	37.04	36.74	37.6	38.06	36.87	Undet.	37.26± 0.55	1.49
10^4	32.26	32.77	32.31	33.94	34.69	33.34	33.22± 0.96	2.90
10^5	29.44	29.11	29.17	29.95	29.89	29.93	29.58± 0.39	1.32
10^6	26.03	25.96	25.64	26.15	26.7	26.42	26.15± 0.37	1.42

C. tropicalis

Log target copies/ PCR reaction	Day 1			Day2			Mean Ct ± SD	% CV
	Ct 1	Ct 2	Ct 3	Ct 1	Ct 2	Ct 3		
10^2	41.19	39.79	38.16	38.88	37.79	38.58	39.07± 1.25	3.19
10^3	35.33	35.45	35.7	35.37	35.45	35.76	35.51± 0.18	0.50
10^4	30.83	30.85	30.68	30.38	30.56	30.73	30.67± 0.18	0.58
10^5	26.96	27.16	27.07	27.15	27.17	27.08	27.10± 0.08	0.29
10^6	23.94	24.08	24	24.08	23.95	24.02	24.01± 0.06	0.25

C. dubliniensis

Log target copies/ PCR reaction	Day 1			Day2			Mean Ct ± SD	% CV
	Ct 1	Ct 2	Ct 3	Ct 1	Ct 2	Ct 3		
10^2	38.1	38.26	37.41	38.73	37.43	38.38	38.05± 0.53	1.40
10^3	34.13	34.04	34.13	34.3	34.39	34.36	34.23± 0.14	0.42
10^4	30.97	31.06	31.09	30.8	30.83	31.01	30.96± 0.12	0.39
10^5	27.45	27.54	27.72	27.56	27.43	27.45	27.53± 0.11	0.40
10^6	23.58	23.46	23.56	23.99	23.9	23.85	23.71± 0.22	0.91

SUPPLEMENTARY FIGURES

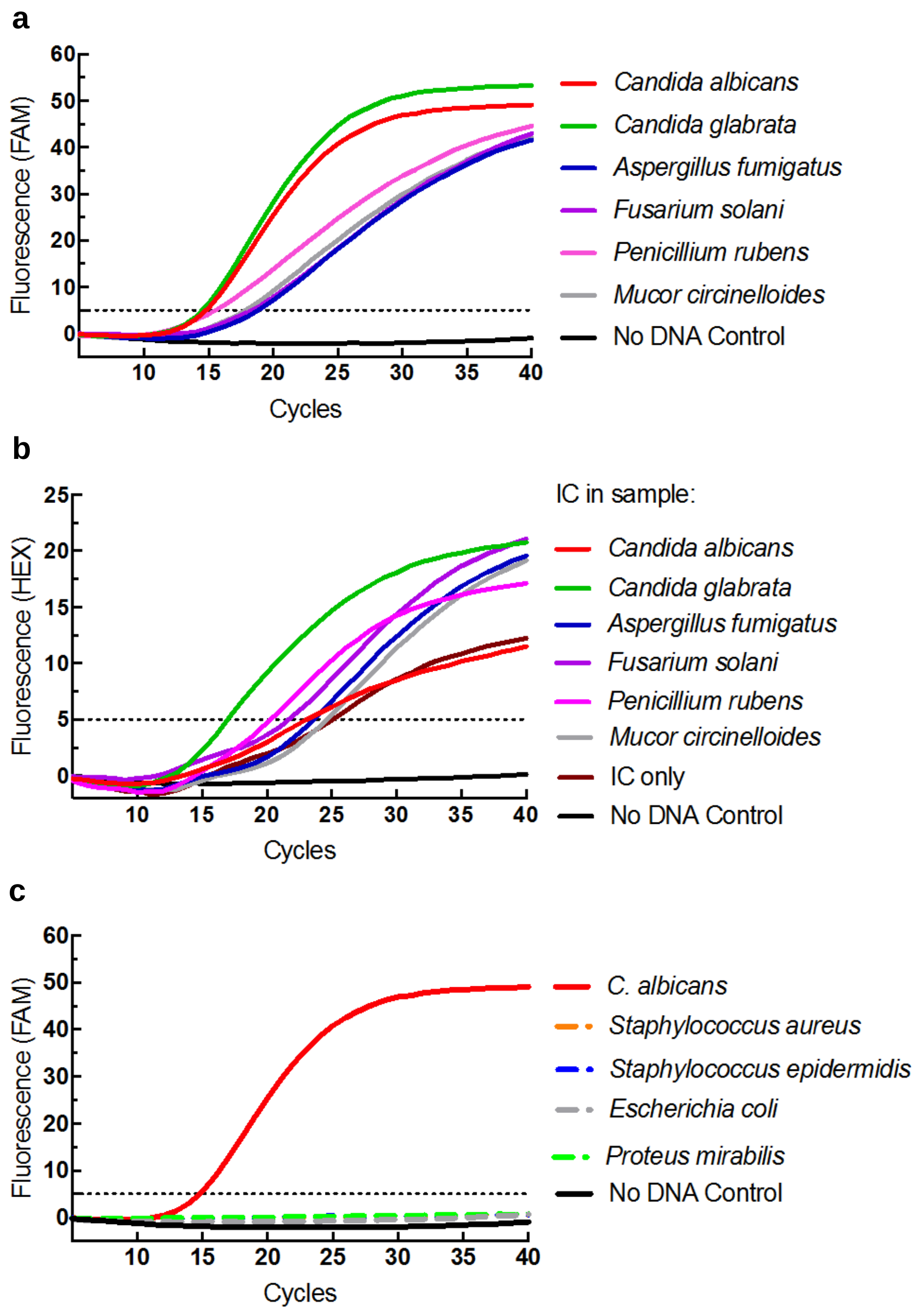

Figure S1. Amplification curves for detection of accumulated amplicons by the pan-fungal probe and internal control probe in FAM and HEX detection channels. (**a**) Demonstration of the amplification curve for detection of several different genera of fungal genomic DNA by

the pan-fungal reaction. (**b**) Amplification curve of internal control template in every sample to monitor amplification activity. (**c**) Functional specificity of pan-fungal probe towards detection of bacterial genomic DNA with *C. albicans* genomic DNA as a positive control.
